# Immunomodulatory Effects of Mesenchymal Stromal Cells in Crohn's Disease

**DOI:** 10.1155/2012/187408

**Published:** 2012-09-20

**Authors:** Ilse Molendijk, Marjolijn Duijvestein, Andrea E. van der Meulen-de Jong, Welmoed K. van Deen, Marloes Swets, Daniel W. Hommes, Hein W. Verspaget

**Affiliations:** ^1^Department of Gastroenterology and Hepatology, Leiden University Medical Center, 2333 ZA Leiden, The Netherlands; ^2^Division of Digestive Diseases, University of California Los Angeles, Los Angeles, CA 90095, USA

## Abstract

The ability of mesenchymal stromal cells (MSCs) to suppress immune responses combined with their potential to actively participate in tissue repair provides a strong rationale for the use of MSCs as a new treatment option in diseases characterized by inflammation and severe tissue damage, such as Crohn's disease (CD) and perianal fistulas. Multiple studies have shown that MSCs suppress a range of immune cells, such as dendritic cells (DC), naïve and effector T cells, and natural killer (NK) cells. Recently published papers attribute the immunosuppressive capacity of MSCs to soluble factors produced by MSCs, such as prostaglandin E2 (PGE_2_), inducible nitric oxide synthase (iNOS), and indoleamine 2,3-dioxygenase (IDO). Promising results are obtained from phase I and II clinical trials with autologous and allogeneic MSCs as treatment for refractory CD and perianal fistulas; however the question remains: what are the molecular mechanisms underlying the immunomodulating properties of MSCs? This paper highlights the present knowledge on the immunosuppressive effects of MSCs and its complexity in relation to CD and perianal fistulas.

## 1. Crohn's Disease

Crohn's disease (CD) and ulcerative colitis (UC), collectively called inflammatory bowel disease (IBD), are chronic diseases characterized by idiopathic inflammation of the gastrointestinal tract. The peak age of onset for CD is 15–30 years, without gender preference [1]. An inappropriate immune response to extracellular pathogens in the gut in a genetically predisposed host, for example, *NOD2/CARD15 *gene associated, is thought to be the cause of IBD [2, 3]. However, the exact etiology remains unclear. The incidence of CD has classically been higher in developed countries; however, urbanization and modernization of undeveloped countries seem to increase the incidence of CD to a similar level as the developed countries [4]. CD can affect any part of the intestine, from mouth to anus, but preferentially involves the ileum and colon. Characteristic for CD are skip lesions; inflamed parts of the intestine interspersed by apparent normal healthy tissue.

Patients with CD can suffer from diarrhea (with blood or mucus), abdominal pain, fever, weight loss, nausea, vomiting, and fatigue [5]. Frequent complications in CD are abscess and stricture formation, intestinal obstruction, and fistulas [2–6]. Fistulas are abnormal connective passages from the epithelial lining of the intestines to another organ or to the skin caused by inflammation. At least one fistula episode (54% perianal) was diagnosed in 35% of CD patients in a large cohort from Minnesota with a follow-up time of 25 years [7]. Patients with perianal fistulas have symptoms like pain, discharge, incontinence, and perineal and genital disfigurement [8]. The diagnosis of CD is established by the clinical features confirmed by endoscopy. Biopsy specimens from inflamed gut mucosa typically show transmural inflammation, including submucosal oedema, ulcerations, and fibrosis.

The choice of medical treatment depends on the location of disease, its severity, and response to earlier therapy. Immunosuppressive therapy (e.g., 6-mercaptopurine, azathioprine, and methotrexate) and biological anti-TNF-*α* therapies (e.g., infliximab and adalimumab) are mostly used to treat active disease and prevent relapses. Unfortunately, 70%–90% of the patients will eventually need surgery during the course of the disease, because the disease responds less to medical therapies over time. Approximately 39% of the patients with CD will even require repeated surgery [9]. An anti-TNF-*α* therapy is the first choice in the treatment of patients with perianal fistulas. However, even with treatment, perianal fistulas often lead to physical and emotional distress and only in 46% of the cases perianal fistulas heal completely [10]. The surgical approach tries to control infectious complications by drainage of abscesses by placement of noncutting silastic setons. Sometimes fecal diversion is needed (stoma) to attenuate perianal symptoms. When these goals have been reached, surgery is aimed to eradicate the fistula while preserving fecal continence. In this latter phase, surgery depends upon the type of fistula and its anatomical extent. Standard surgical approaches are fistulotomy or a mucosal advancement plasty, which are unsuccessful in over 50% of the cases [11]. Effective medical therapeutics for patients with CD and perianal fistulas, refractory to or dependent on the conventional strategies, are needed. 

To investigate new therapeutic options for CD, two mouse models are most commonly used to induce colitis [12, 13]. In the most frequently used model, dextran sulfate sodium (DSS) polymers are added to drinking water for several days. This is directly toxic to gut epithelial cells of the basal crypts and affects the integrity of the mucosal barrier. This acute colitis is characterized by ulcerations, bloody diarrhea, and infiltration with granulocytes and is particularly useful to study the contribution of innate immune mechanisms to colitis [12, 13]. Colitis can also be induced by intrarectal administration of the haptenizing agent 2,4,6-trinitrobenzene sulfonic acid (TNBS) diluted in ethanol [12–14]. Ethanol is necessary to break down the mucosal barrier, whereas TNBS haptenizes the microbial flora in the colon in order to stimulate an immune response. This model is useful to study T helper cell-dependent mucosal immune responses since CD4+ T cells have been shown to play a key role in TNBS colitis [13].

## 2. Mesenchymal Stromal Cells 

In addition to hematopoietic stem cells, the bone marrow also contains mesenchymal stromal cells (MSCs) [15]. These MSCs are multipotent cells, capable of differentiating into multiple lineages of the mesenchyme, including osteoblasts, adipocytes, and chondroblasts [16–18]. Apart from the bone marrow, MSCs have been isolated from several other tissues, such as adipose tissue [19], peripheral blood [20], umbilical cord blood [21], and placenta [22]. Recently MSCs have also been isolated from gingiva (gMSCs), a unique oral tissue attached to the alveolar bone of tooth sockets [23]. Not only is the oral cavity easily accessible, but gMSCs can also be easily obtained from discarded tissue from routine dental procedures. According to the minimal criteria proposed by the International Society for Cellular Therapy [24], MSCs should be identified based on their ability to adhere to plastic in standard culture conditions and on their ability to differentiate *in vitro* into bone, fat, and cartilage. They must express CD73, CD90, and CD105 and may not express CD11b or CD14, CD19, CD45, CD79*α*, and HLA-DR surface molecules. These MSCs possess a unique combination of functional activities, that is, they have the ability to suppress immune responses and they are able to actively participate in tissue repair processes. The ability of MSCs to suppress immune responses could be relevant in the treatment of luminal CD, whereas in the case of perianal fistulas their potential to repair tissue is thought to be more important [25]. In addition, MSCs are immunologically relatively inert since they are poor antigen presenting cells (APCs) and do not express MHC class II or costimulatory molecules. In accordance, expanded MSCs do not stimulate T-cell proliferation in mixed lymphocyte reactions (MLRs) and are also able to downregulate alloreactive T-cell responses when added to mixed lymphocyte cultures [18, 26–29]. These findings suggest that allogeneic MSCs could be very useful in the clinic and their expansion potential provides the possibility to generate a stock with “off the shelf-”treatment potential. However, in contrast to the general idea that MSCs are not immunogenic, Nauta et al. showed that multiple injections of allogeneic MSCs after bone marrow transplantation in sublethally irradiated mice decrease engraftment whereas syngeneic MSCs promote engraftment [30]. In addition, allogeneic MSCs are capable of inducing a memory T-cell response in immunocompetent hosts. Although further studies are needed to elucidate the situations wherein MSCs can be immunogenic, these findings should be taken into account when allogeneic MSCs are used in clinical setting. Furthermore, MSCs have been shown to alter cytokine secretion profiles of dendritic cells (DC), naïve and effector T cells, and natural killer (NK) cells, which is accompanied by the induction of a more anti-inflammatory or tolerant phenotype [31]. 

## 3. MSCs and Antigen Presenting Cells

Allogeneic human MSCs (hMSCs) have a reversible inhibitory effect on the differentiation of monocytes into DCs [32–34] and are able to downregulate the expression of the costimulatory molecules CD80 and CD86 in the case of mature DCs. DCs that are cocultured with MSCs before adding them to T cells show a reduced ability to activate these T cells to proliferate [32]. When hMSCs are cocultured with unmatched peripheral blood mononuclear cells (PBMCs), hMSCs reduce T-cell proliferation and the secretion of interferon-gamma (IFN-*γ*) by these cells, which is normally induced upon coculture with allogeneic cells [27, 33]. In addition, IFN-*γ* secretion and T-cell proliferation can be partially restored when lipopolysaccharides (LPS) or anti-CD40 monoclonal antibodies, which both promote APC-maturation, are added to cocultures with hMSCs. This suggests that hMSCs may have an effect on normal APC maturation [33]. Furthermore, Aggarwal and Pittenger [31] showed a significant decrease of 50% in TNF-*α* secretion in response to LPS when type 1 DCs are cocultured with hMSCs. Upon LPS stimulation, type 2 DCs secrete moderate levels of IL-10, an anti-inflammatory cytokine. Interestingly, when cocultured with hMSCs, the percentage of IL-10 increases with 140%. These results suggest that hMSCs cocultured with matured DCs provide a more anti-inflammatory milieu *in vitro.*

## 4. MSCs and T Cells

In the same paper, Aggarwal and Pittenger [31] describe the interaction between MSCs and T cells. Naïve T cells were activated to differentiate into T helper cell type 1 (Th1) or T helper cell type 2 (Th2) in the presence or absence of hMSCs. A significant decrease of 60% in levels of IFN-*γ* was seen when hMSCs were present during the differentiation into Th1 cells compared to differentiation without hMSCs. Moderate levels of IL-4 were secreted during the differentiation of naïve T cells into Th2 cells, a cytokine known to induce this T-cell differentiation. The average increase in the amount of IL-4 in the presence of hMSCs during this differentiation process was 500%, suggesting that hMSCs provide significant help for naïve T cells to differentiate into Th2 cells.

Anergic T cells express the IL-2 receptor, but do not proliferate or produce IL-2 in response to adequate antigenic stimulation. Normally, this anergic state can be abolished by the addition of exogenous IL-2. However, MSCs can induce a anergic state in T cells that is only partially reversible, also known as a split anergic state [35, 36]. Glennie et al. [35] showed that after addition of IL-2 and removal of MSCs, IFN-*γ* production was restored; however, the proliferation of PBMCs was not.

A naturally anergic and suppressive T-cell population that induces immunologic self-tolerance and plays a key role in the development of autoimmune diseases, such as IBD, are CD25+CD4+ cells [37]. Maccario et al. [38] demonstrated a large increase in the number of CD4+CTLA-4+ and CD4+CD25+CTLA-4+ cells in the presence of third-party MSCs and a slight increase in these subsets in the presence of autologous MSCs compared to control. CTLA-4 is a T cell inhibitory receptor involved in mediating T cell anergy and tolerance [39]. These observations indicate that MSCs increase the levels of a regulatory T cell (Treg) phenotype which suppresses Th1 immune responses. In a TNBS model, systemic infusion of adipose-derived MSCs (adMSCs) has been reported to ameliorate the clinical and histopathologic severity of TNBS colitis [40]. This therapeutic effect was found to be mediated by down-regulating TNF-*α*, IFN-*γ*, IL-1*β*, IL-6, and IL-12 and on the other hand elevating regulatory cytokine IL-10 in colonic tissue. A second paper from the same group [41] corroborated these data by showing that systemic infusion of adMSCs protects against experimental DSS colitis and sepsis. An increased number of FoxP3+ was not only described in colon tissue but also in mesenteric lymph nodes (MLNs) [42]. Mice intravenously treated with bone marrow-derived MSCs (bmMSCs) after induction of colitis with TNBS had an approximately 2.1-fold higher absolute number of CD25+FoxP3+ cells compared to control mice.

Besides Th1 and Th2 cells, a subset of Th cells that produces high levels of IL-17 exists. These so-called Th17 cells protect against extracellular pathogens at mucosal surfaces and are thought to play an important role in inflammation and tissue damage in autoimmune diseases such as CD. During the differentiation of naïve T cells into Th17 cells, the presence of MSCs inhibits the production of inflammatory cytokines and slightly induces the production of IL-10 and concomitantly strongly enhances the expression of FoxP3 mRNA levels. Additionally, the presence of MSCs in a culture with stimulated fully differentiated Th17 cells results in decreased levels of IL-17 and IL-22, increased IL-10 production, and, again, enhanced FoxP3 mRNA levels. The induction of FoxP3 mRNA expression gives rise to a functional Treg phenotype, which is confirmed by the observed ability of Th17 cells to inhibit proliferation of T cells during coculture with MSCs. In contrast, Th17 cells that have not been in contact with MSCs do not have an effect on the proliferation of T cells [43]. Liang et al. [44] revealed that human umbilical cord MSCs (ucMSCs) in coculture can significantly inhibit IL-17 production by lamina propria mononuclear cells and splenocytes. In a TNBS model, these *in vitro* results were confirmed, that is, ucMSC-treated mice had significantly lower levels of IFN-*γ*, IL-6, IL-17, and IL-23 in the colon compared to the control mice.

## 5. MSCs and NK Cells

NK cells are cytotoxic effector cells of the innate immune system that play a key role in the elimination of virally infected or transformed cells. Upon stimulation with IL-2, purified NK cells produce IFN-*γ* and when these stimulated NK cells are subsequently cocultured with hMSCs, the levels of secreted IFN-*γ* decreases with 80% [31]. Furthermore, allogeneic MSCs inhibit IL-2- and IL-15-induced proliferation of resting NK cells. However, they seem to have only a partial inhibitory effect on already proliferating NK cells. Intriguingly, autologous and allogeneic NK cells stimulated with IL-2, but not freshly isolated NK cells, show a strong cytolytic activity against MSCs. MSC pretreated with IFN-*γ*, however, are less susceptible to lysis by the NK cells [45].

Although MSCs are able to alter cytokine secretion profiles of different immune cells in order to induce a more anti-inflammatory or tolerant phenotype, the question remains: what are the mechanisms by which MSCs exert this biological activity? Several studies suggest that MSC-derived soluble factors may contribute to this induced immunosuppression [26, 29].

## 6. Prostaglandin E2 (PGE_2_)

PGE_2_ is one of the immunosuppressive molecules produced by MSCs when activated by inflammatory cytokines such as IFN-*γ* and TNF-*α* [31]. Non-bone marrow-derived lamina propria stromal cells constitutively produce COX-2-dependent PGE_2_, as shown by Newberry et al. [46], which suggests that the expression of inflammatory mediator COX-2 by lamina propria stromal cells contributes to the low immune response against antigens in the mucosa of the small intestine. The ability of MSCs to inhibit Th17 differentiation and to induce a regulatory phenotype is strongly believed to be mediated by the COX-2-dependent soluble factor PGE_2_ (Duffy et al. [47]). In an experimental arthritis model, Bouffi et al. [48] demonstrated that MSCs do not only have a local immunosuppressive effect but also a systemic are. Secreted PGE_2_ was the main factor in reducing the inflammation locally whereas systemic immune suppression by MSCs was mediated by the switch of the inflammatory Th1/Th17 profile towards a more Th2 response. Also in the amelioration of TNBS-induced colitis, the importance of PGE_2_ produced by human adMSCs was demonstrated [40]. Furthermore, in addition to a therapeutic efficacy of bmMSCs, Tanaka et al. [49] showed in a DSS model a decrease in mRNA expression of inflammatory mediators TNF-*α*, IL-1*β*, and COX-2 in the rectum of rats treated with bmMSCs compared to control values, again suggesting that this might be a pivotal mechanism explaining the immunosuppressive effects of MSCs.

## 7. Inducible Nitric Oxide Synthase (iNOS) and Indoleamine 2,3-Dioxygenase (IDO) 

Nitric oxide (NO) is an important signaling molecule and is involved in tissue homeostasis and immunoregulatory functions. iNOS is expressed by murine MSCs as a result of stimulation with IFN-*γ* combined with TNF-*α*, IL-1*α*, or IL-1*β* [50]. Immunosuppression is achieved when high levels of NO are released, but no immunosuppression is seen with MSCs derived from INOS^−/−^ or IFN*γ*R1^−/−^ mice. In addition, only wild-type MSCs but not iNOS^−/−^- or IFN*γ*R1^−/−^-MSCs prevented graft-versus-host disease and delayed-type hypersensitivity in mice. Interestingly, iNOS^−/−^-MSCs even worsened this delayed-type hypersensitivity. These results suggest that the combination of iNOS and IFN-*γ* is crucial to achieve immunosuppression [50]. Substantiating this theory, a recently published paper on the therapeutic effect of aspirin in TNBS-induced colitis [51] demonstrated that NO-releasing aspirin, in contrast to regular aspirin, COX-1 inhibitors and SC-560, accelerated colonic healing characterized by a down-regulation of COX-2, iNOS, IL-1*β*, and TNF-*α* mRNAs.

The mechanism of MSC-mediated immunosuppression is different in mice and humans. While murine MSCs use predominantly iNOS to control immune responses, human MSCs mainly utilize indoleamine 2,3-dioxygenase (IDO), an immunoregulatory enzyme regulating tryptophan levels, and express only very low levels of iNOS [52]. However, both mouse MSCs and human MSCs need IFN-*γ*, combined with TNF-*α*, IL-1*α*, or IL-1*β*, in the induction of the suppression of immune cells such as T cells and NK cells through these enzymes [53, 54]. 

In addition, to inhibit T-cell proliferation, MSCs might have to be in contact with the T cells, as shown by Ren et al. [50, 52], since this inhibition was abolished when these cells were separated by transwells. Therefore, MSCs express T-cell specific chemokines to attract immune cells and may thereby effectuate their immune response through soluble factors.

Recently, gMSCs were found to suppress PBMC proliferation in the same manner as bmMSCs by Zhang et al. [23] Comparable to bmMSCs, gMSCs induce several immunosuppressive factors in response to IFN-*γ*, including IL-10, COX-2, iNOS, and IDO. IDO and IL-10, but not COX-2 or iNOS, which were upregulated by IFN-*γ*, contributed to the gMSC-mediated suppression of PBMC proliferation. A protective effect of gMSCs in a DSS model with C57BL/6 mice was also observed, exemplified by the fact that gMSCs significantly suppressed mucosal ulceration of the colon, ameliorated transmural inflammation, and decreased wall thickness, thereby restoring the colonic tissue homeostasis. In the nontreated DSS mice, an infiltration of CD4+ cells was seen in the mucosal and muscularis layers and an increased expression of IL-6, IFN-*γ*, and IL-17 was found in the inflamed colons. Systemic infusion of gMSCs was found to diminish the amount of CD4+ cells in the colon and to decrease the levels of inflammatory cytokines at the intestinal mucosa. In addition, anti-inflammatory cytokine IL-10 levels were increased and an influx of Tregs was seen.

Our group [55] demonstrated that IFN-*γ* activation of MSCs (iMSCs) before administration of these MSCs to mice enhanced their capacity to inhibit Th1 inflammatory responses. Stimulation of human MSCs with IFN-*γ* increased their expression of IDO and activation of mouse MSC with this cytokine induced higher iNOS levels. In a DSS model, we showed that mice treated with human iMSCs have a less severe mucosal mononuclear cell infiltration and reduced disruption of crypt architecture, compared to mice treated with nonprestimulated MSCs. In addition, BALB/c mice treated with mouse iMSCs after the induction of colitis with TNBS gained 2,5% body weight at sacrifice compared to a decrease in body weight of 1,3% in nonactivated MSC-treated animals and even 4,9% in the PBS group. In both experimental colitis models, TNF-*α* and IL-6 levels were elevated but treatment with iMSCs was accompanied by a strong reduction in the amount of TNF-*α* and IL-6 in the colon.

## 8. Clinical Trials on MSC Treatment of Luminal and Fistulizing CD

Several papers on clinical trials using MSCs as treatment for CD and perianal fistulas have been published [56–59]. All these studies demonstrated that the administration of autologous or allogeneic MSCs is safe and feasible and some studies have suggested a potential therapeutic effect of these cells. In our hands [56], three out of nine patients with luminal CD treated with autologous bmMSCs showed clinical response, defined as a decrease of 70 or more points in CD Activity Index (CDAI), 6 weeks after MSC infusion. However, also three out of nine patients required surgery 7, 12, and 14 weeks, respectively, after MSC injection due to disease worsening. In order to assess mucosal changes, biopsies of inflamed colonic mucosa were taken at week 0 and week 6 after MSC infusion. A trend of lower CD4+ T cells and higher CD4+CD127+ regulatory T cells was observed in the biopsies taken at week 6 compared to the biopsies at week 0. *In vitro,* the proliferation of PBMCs in the presence of autologous bmMSCs derived from patients with CD was reduced in a cell dose-dependent manner similar to bmMSCs from healthy donors, indicating a normal immunosuppressive activity of the CD patient-derived MSCs. Moreover, in the presence of immunosuppressive drugs such as azathioprine, methotrexate, 6-mercaptopurine, and infliximab, MSCs maintained their multilineage differentiation potential and their ability to suppress proliferation of PBMCs *in vitro* was not altered. In addition, the neutralization of TNF-*α* by the anti-TNF-*α* agent infliximab did not hamper the suppressive effect of MSCs [60]. Apparently, an optimal treatment protocol of luminal CD has not been achieved yet but comedication seems not to affect the functional activity of the MSCs.

Encouraging results have been obtained from phase I and II trials in patients with fistulizing CD. In one study, a total of nine fistulas in four patients were injected with adMSCs to demonstrate the safety of the local application of MSCs in fistulizing CD [57]. Although the study was not designed to determine effectiveness, 75 percent of the fistulas treated with adMSCs were considered healed after eight weeks and no adverse events were observed. This trial was followed by a phase II study, sponsored by Cellerix, to evaluate the efficacy and safety of adMSCs treatment in 49 adult patients with complex perianal fistulas from cryptoglandular disease (*n* = 35) or CD (*n* = 14) [58]. Patients were randomly assigned to either intralesional treatment with fibrin glue alone or with fibrin glue plus 20 million adMSCs. When the fistulas were not healed after eight weeks, a second dose of fibrin glue or 40 million adMSCs plus fibrin glue was injected. In four out of the 25 patients (16%) who received fibrin glue without MSCs, healing of fistulas was observed compared to 17 out of the 24 patients (71%) who received fibrin glue plus adMSCs. In both the cryptoglandular disease and the fistulizing CD group efficacy was observed. In another trial, 10 patients refractory to or unsuitable for current available therapies for fistulizing CD were locally injected with autologous bmMSCs at four-week intervals [59]. Each patient underwent a median of four injections with approximately 20 million bmMSCs per time as long as autologous bmMSCs were available. In all patients, an improvement of CDAI and perianal disease activity index (PDAI) was observed and rectal mucosal healing was induced. In addition, fistulas closed completely in seven out of the ten patients without any adverse events.

Although only one clinical trial on MSCs for the treatment of luminal CD has been published, a discrepancy in efficacy between systemic infusion of MSCs for luminal CD and local injection for the treatment of fistulizing CD might be observed. It is thought that after intravenous administration, MSCs migrate to the site of disease to modulate immune responses and contribute to the tissue repair. In an experimental colitis model, Gonzalez-Rey et al. [41] showed homing of intraperitoneal injected human adMSCs to inflamed colon, but not to noninflamed colon. In addition, Duijvestein et al. [55] observed this specific homing using human bm-iMSCs. However, iMSCs were found across a wide array of tissues and only a small proportion of iMSCs was detected in the diseased intestine, suggesting that may be not all cells survive and that additional mechanisms such as soluble factors might contribute to the immunosuppression. One could speculate that compared to systemic infusion, higher numbers of cells reach the damaged tissue when injected locally and therefore are more likely to perform their immunosuppressive and tissue regenerative functions directly or via soluble factors. Randomized controlled trials with a sufficient number of patients are needed in order to prove the actual efficacy of MSCs in the treatment of luminal and fistulizing CD.

## 9. MSCs and Their Immunomodulatory Complexity

Although encouraging results from clinical trials on fistulizing CD have been published, some reservations should be made since similar phase III studies in patients with severe refractory intestinal graft-versus-host disease (GvHD) demonstrated no effectiveness of MSC treatment as opposed to the initial phase II studies [61]. This lack of efficacy might be procedural since François et al. [62] indicated that cryopreserved MSCs, used in clinical trials, could not suppress T-cell proliferation *in vitro *after thawing. However, after 24 hours of culture, the immunosuppressive effect of these postthaw MSCs was restored to a level comparable to MSCs cultured for 7 days. In addition, IFN-*γ* stimulated freshly thawed MSCs expressed very low levels of IDO compared to cultured MSCs, which might be an explanation for the diminished immunosuppressive properties of these MSCs. Although in clinical trials mostly MSCs passages 1 or 2 are used, MSCs do lose their typical spindle shape morphology and their proliferation rate seems to decrease with increasing passage number. MSCs from younger donors seem to maintain these characteristics up to higher passages then MSCs from older donors [63–65]. Furthermore, in a recently published paper, Li et al. [66] showed that MSCs have the capacity to promote immune responses instead of suppressing them. MSCs were cocultured with freshly isolated splenocytes in the presence of anti-CD3 to stimulate the latter to produce IFN-*γ* and TNF-*α*. At high splenocyte, MSC ratios T-cell proliferation was inhibited, whereas at lower densities this proliferation was found to be enhanced. This suggests that depending on the relative concentration of T cells and MSCs, MSCs can either have an immunosuppressive or an immunostimulatory effect. Remarkably, MSCs enhanced T cell proliferation when splenocytes were stimulated with low concentrations of anti-CD3, which indicates that insufficient levels of inflammatory cytokines most probably caused this effect. Interestingly, MSCs pretreated with IFN-*γ* and TNF-*α* before cocultring them with splenocytes inhibited T-cell proliferation regardless of the number of T cells present. This clearly indicates that the level of proinflammatory cytokines determines whether MSCs act as immune suppressors or enhancers. Furthermore, iNOS^−/−^ MSCs cocultured with splenocytes were not only unable to suppress T-cell proliferation, they even caused a dramatic elevation of the immune response. Intriguingly, IFN-*γ* and TNF-*α* pretreatment of iNOS^−/−^ MSCs also enhanced the proliferation of T cells, once more illustrating the complexity of MSC-mediated immunomodulation. These findings are of high importance when considering MSCs as a new treatment option, especially in diseases with an alternating severity of inflammation such as CD. MSCs may switch from immunosuppressive cells to immunoenhancing cells in the absence of sufficiently high levels of proinflammatory cytokines, which could possibly lead to a flareup instead of a complete remission in patients with a mild course of CD. Although the neutralization of TNF-*α* with the anti-TNF-*α* agent infliximab did not hamper the suppressive effect of MSCs* in vitro* [60], nothing is known about the effect of anti-TNF-*α* agents on MSCs *in vivo*.

## 10. Concluding Remarks

The ability of MSCs to suppress immune responses, particularly T-cell proliferation, combined with their potential to actively participate in tissue repair provides a strong rationale for the use of MSCs as a new treatment option in diseases characterized by inflammation and severe tissue damage, such as CD. Several studies have shown the ability of MSCs to alter cytokine secretion profiles of DCs, naïve and effector T cells, and NK cells to induce an anti-inflammatory or regulatory milieu *in vitro *and* in vivo*. However, recently published papers attribute the immunosuppressive capacity of MSCs to soluble factors produced by MSCs when stimulated by IFN-*γ* or TNF-*α*, such as PGE_2_, iNOS, and IDO. 

The fact that MSCs are poor APCs and do not express MHC class II or costimulatory molecules in a resting state suggests that allogeneic MSCs could be used for clinical applications. In addition, the possibility to use allogeneic MSCs gives rise to a possible stock production with “off the shelf-”treatment potential. Several clinical studies have been performed using both autologous and allogeneic MSCs as a treatment for patients with luminal CD or perianal fistulas. Although encouraging results are obtained from these phase I and II trials, the discouraging results of two phase III studies with MSCs in severe refractory intestinal GvHD are a serious drawback. Further research into the molecular mechanisms through which MSCs act is necessary to understand the complexity of the immunomodulatory effects of MSCs and to develop a new treatment modality for patients with refractory CD and/or perianal fistulas.
